# The impact of practical training based on narrative nursing on the humanistic care ability of Chinese intern nurses

**DOI:** 10.3389/fmed.2025.1635846

**Published:** 2025-11-07

**Authors:** Lili Dai, Yuqin Han, Qunhua Jiang, Wenxian Ge, Hong Zhao, Ping Xu

**Affiliations:** 1 Department of Orthopedics, Shanghai Fengxian District Central Hospital, Shanghai, China; 2 Department of Nursing, Shanghai Fengxian District Central Hospital, Shanghai, China

**Keywords:** clinical intern nurses, practical training, narrative nursing, humanistic care, empathy, China

## Abstract

**Objective:**

Improving the humanistic care ability of intern nurses and fostering a deep-seated sense of compassion are crucial goals. In this study, we aimed to investigate the effect of practical training based on narrative nursing on the humanistic care ability of intern nurses.

**Methods:**

Retrospective analysis was conducted on intern nurses who received practical training based on narrative nursing (*n* = 70) or regular training (*n* = 70) for 6 months. The differences in humanistic care ability, empathy, professional values, professional identity, and satisfaction between the two groups were evaluated. The univariate logistic regression analysis was constructed to determine the influencing factors of humanistic care ability.

**Results:**

Compared to those who received regular training, intern nurses who received practical training based on narrative nursing showed significant improvements in humanistic care ability, empathy, professional values, and professional identity (all *p* < 0.05). Additionally, intern nurses were highly satisfied with practical training based on narrative nursing. Univariate logistic regression analysis revealed that practical training based on narrative nursing was an influencing factor of humanistic care ability (*p* < 0.05).

**Conclusion:**

Practical training based on narrative nursing can significantly improve the humanistic care ability, empathy ability, professional values, and professional identity of nursing interns.

## 1 Introduction

The resurgence of the integration of medicine and the humanities is increasing in contemporary society. The main objectives of current nursing practice involve improving the humanistic care ability of clinical nurses and fostering a deep-seated sense of compassion and awareness (1). Considering the evolving modern medical paradigm, nursing professionals must possess not only adept clinical skills but also essential humane attributes (2). Fostering the capacity for humanistic care among nurses during their internship is crucial for establishing a solid groundwork for them to embrace a “patient-centered” approach when they become professional nurses (3, 4). As an important reserve force in the nursing field, humanistic care ability directly affects the quality of future nursing services of intern nurses and the overall quality of the nursing team. A multicenter cross-sectional study recruiting nurses from tertiary and secondary hospitals in China revealed that nurses have comparatively modest proficiency in humanistic care ability (5). Additionally, there are few clinical practice studies on the humanistic care ability of intern nurses in China, and there is no practical model guided by specific theories for reference (6). Therefore, nursing managers should expand the scope of humanistic care practices, continue to provide professional training, and improve the humanistic care ability of intern nurses.

Narrative nursing is a novel form of humanistic care intervention that integrates the principles and techniques of narrative medicine or narrative therapy into nursing practice. It has garnered significant interest in the realm of healthcare in recent years (7). Narrative nursing uses various information media (such as literature, pictures, film, and television) as the carrier. Through communication, discussion, and analysis in teaching activities, the deeper educational messages behind the story are explored to foster a sense of humanistic care (8, 9). Narrative nursing aims at focusing on the patient and urging individuals to share their perspectives and thoughts on their health by recounting personal stories and experiences (10). By using narrative techniques, nurses can gain important insights into the suffering of patients and obtain accurate information on their medical status and emotional well-being, thereby increasing their ability to provide compassionate care (11). This approach also helps foster strong bonds between nurses and patients and enhances the delivery of healthcare services (12, 13). By engaging in hands-on exercises involving narrative nursing, such as reading, writing, and reflective thinking, nurses can gain a better understanding of the circumstances of the patients (14). Using the stories of 73 Chinese Nightingale Prize winners as narrative education materials in the course of humanistic culture, Chang et al. reported that compared to students who did not attend the course of humanistic care, narrative nursing effectively improved the humanistic care literacy of undergraduate nursing students (15). Through lectures, group discussions, questions and answers, and PowerPoint presentations, a recent control study revealed that narrative medicine training significantly improved the total empathy score and the total humanistic care quality score of new nurses in cancer hospitals (16). Although the scope and depth of the utilization of narrative education in nursing instruction is being improved and updated continuously, clinical nurses still find it difficult to understand narrative nursing concepts, mostly due to the lack of structured theoretical frameworks and guidelines for clinical practice (17). Consequently, nurses cannot confidently apply humanistic care knowledge in their daily practice (18). Moreover, few empirical studies have examined the influence of humanistic care training on clinical intern nurses in China.

Considering the insufficient research on the impact of practical training based on narrative nursing on the humanistic care ability, empathy ability, professional values, and professional identity of nursing interns in China, in this study, we investigated the effectiveness of implementing narrative nursing-based practical training on nursing interns to better determine the relationship between narrative nursing and humanistic care ability.

## 2 Methods

### 2.1 Research objects

**Figure 1:**
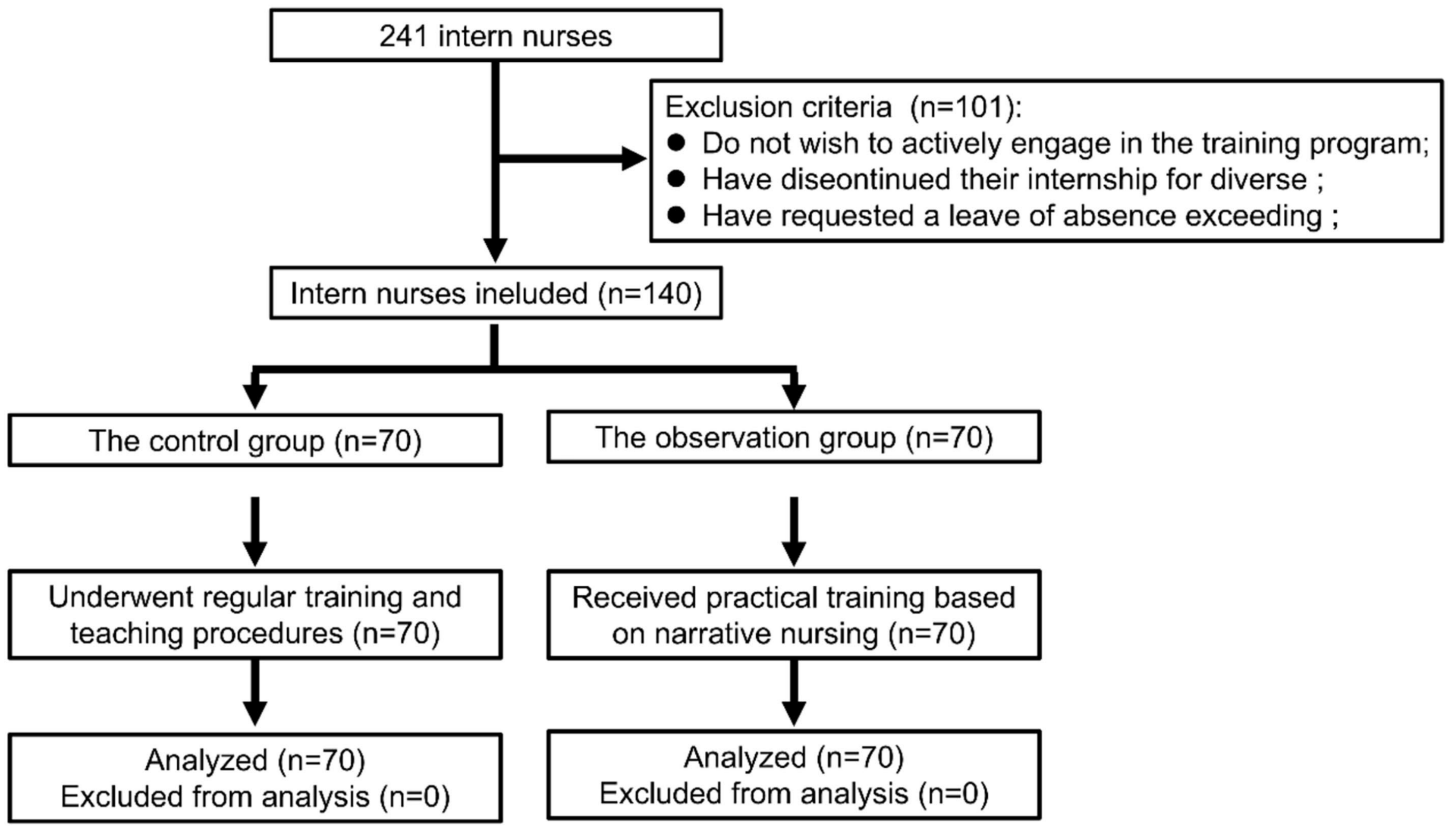
Flow diagram illustrating enrollment and training process of intern nurses.

This retrospective study included nursing interns who received training at Fengxian District Central Hospital from May 2022 to May 2024. Nursing interns were divided into a control group or an observation group based on the training plan. A flow chart of the study design is provided in Figure 1.

### 2.2 Inclusion and exclusion criteria

The inclusion criteria were as follows: (1) the participants were intern nurses trained at Fengxian District Central Hospital with an intended internship duration exceeding 8 months; (2) the participants were 18 years old or older; (3) the participants did not have any cognitive behavioral disorders; and (4) the participants provided voluntary consent to participate in the study. The exclusion criteria were as follows: (1) intern nurses who did not wish to actively engage in the training program; (2) intern nurses who discontinued their internship for various reasons; and (3) those who requested a leave of absence exceeding 2 months due to exceptional circumstances during the intervention, rendering them unable to further participate in the study.

### 2.3 Training procedure

#### 2.3.1 Establishing a team of instructional staff

The instructional staff comprised one nursing department head, six specialists in clinical nursing management, four clinical educators, and one graduate student, with 12 members in total. The clinical nursing management specialists had over 20 years of experience in clinical education, held deputy senior or higher professional titles, and were actively involved in clinical nursing, medical humanities, and research. The qualifications for teacher training included (1) holding a bachelor’s degree or higher; (2) possessing a nursing degree or higher professional title; (3) demonstrating proficiency in humanistic care literacy; (4) having over 5 years of experience in clinical teaching or general teaching; and (5) completing training in humanistic care.

#### 2.3.2 Training program for the control group

The training program for the control group included elements from the annual internship teaching plan of the hospital nursing department related to humanistic care. This involved the administration and coordination of humanistic care in hospital nursing, as well as aspects such as the physical environment, facilities, training in humanistic care, patient-focused humanistic care, and nurturing humanistic care ability among nurses. Additionally, the training regimen focused on stressing the importance of humanistic care for clinical educators, fostering the communication skills of interns, and enhancing their capacity for humanistic care. Training sessions were conducted once a week for 60 min per session, spanning 6 weeks for each department, for four departments (internal medicine, surgery, gynecology, and emergency departments). The training program was conducted for 6 months.

#### 2.3.3 Training program for the observation group

The observation group received training in narrative nursing education, which was developed using the training modules completed by the control group. The training was conducted by dividing the units into internal medicine, surgery, gynecology, and emergency departments, using both online and offline learning methods. Using Li Chun’s narrative nursing and IF nursing temperature as key learning resources (19, 20), various training formats were implemented over 6 weeks for each department. To prepare for the training, the narrative material videos corresponding to the teaching content were uploaded on the nursing assistant platform 1 week before the training commenced. The intern nurses were asked to watch the videos and reflect on the guiding questions. The initial 2 weeks involved listening to lectures and watching videos, followed by presenting material stories and situational simulations in weeks 3 and 4. Weeks 5 and 6 were dedicated to hosting reading salons and encouraging diary reflections, as outlined in Table 1. The training course lasted 6 weeks in each department, including four departments (internal medicine, surgery, gynecology, and emergency), for 6 months.

**Table 1:** Content of narrative ability training for nursing students in clinical practice.

Time	Training form	Training frequency	Training content
Week 1–2	Listen to lectures and watch videos	60 min/time, once a week	(1) Nursing professionals provide lectures on the concept of “embracing warmth in nursing through narrative techniques.” (2) Arrange for newly enrolled nursing students to watch the video “Exploring the Role of Temperature in Nursing.”
Week 3–4	Present material stories, situational simulations	60 min/time, once a week	(1) Based on the narrative materials provided by nursing assistants, themes such as “Angelic presence,” “Supporting the chronically ill,” “Compassionate care and communication by emergency and critical care nurses,” and reflections on aging are explored by trainee nursing students in groups of 5–6. They are expected to thoroughly analyze the humanistic care aspects depicted in the stories with no restrictions on the presentation format. Teaching faculty will evaluate and show the most compelling group videos for classroom viewing. (2) Using the clinical case as a foundation, the nursing students in training groups are encouraged to center their narrative nursing discussions around the life of the patient being cared for. By providing holistic care to the patients and applying theoretical knowledge to practical scenarios, the students develop scripts tailored to specific clinical contexts. They implement psychological nursing techniques to effectively capture the emotions and psychological states of each character involved. Emphasis is placed on fostering effective communication and interactions among the roles portrayed by the interns, including doctors, nurses, patients, and family members, to comprehend the narratives underlying the illnesses. This process aims to cultivate empathy among the students and show the importance of humane nursing care. Collaborative discussions and analysis between teachers and students aim to address challenges and culminate in a comprehensive summary provided by the instructors.
Week 5–6	Reading salon, reflective diary	60 min/time, once a week	(1) Organize a literary gathering for sharing narrative tales. (2) Compose a reflective journal in collaboration with the internship department. Nursing trainees must delve deep into the emotions and thoughts of patients, applying their narrative expertise to comprehend both the triumphs and tribulations of the patients’ journey with illness. They should show empathy toward the experiences of the patients and authentically document their emotions. By reviewing the reflective journals of trainee nurses, insights into their psychological well-being and caregiving skills can be gained, facilitating tailored guidance and support.

### 2.4 Data collection

#### 2.4.1 General information

The basic data registered before the training of intern nurses were retrieved. The basic data included gender, age, educational background (ranging from junior high school to graduate level), family residence (local or remote), sibling status, presence of healthcare personnel in the family, previous experience as nursing cadres during college, and motivations for selecting the nursing major. After the training, nursing interns received the questionnaire survey. The questionnaire survey included Caring Ability Inventory (CAI), Jefferson Scale of Empathy (JSE), Nurse Professional Values Scale (NPVS), Professional Identity Scale for Nursing Students (PISNS), and the opinions of intern nurses on various training programs.

#### 2.4.2 Humanistic care ability scores

The humanistic care ability scores of participants were collected. Caring Ability Inventory (CAI) developed by Nkongho is used to investigate intern nurses’ humanistic care competencies (21, 22). This scale comprises of 37 items (Mutual care between good friends, easy understanding of others, etc.) categorized into three dimensions: understanding, courage, and patience, with 14 items in the understanding dimension, 13 items in the courage dimension, and 10 items in the patience dimension. Each item was rated on a 7-point scale ranging from “completely disagree” to “strongly agree,” with 13 items being reverse scored. The scores demonstrated a positive association with the caring ability. The total score for the scale was 37–259. A total score greater than 220.30 indicates a higher level of humanistic care ability, while a score lower than 203.10 suggests a lower level of humanistic care ability, and a score falling between 203.10 and 220.30 indicates a moderate level. The *Cronbach’s α* coefficient for the scale was 0.712, and the split-half reliability was 0.803.

#### 2.4.3 Empathy ability scores

The scores of empathy ability were collected. The empathy ability was measured using the Jefferson Scale of Empathy (JSE) (23), comprising 20 items (I belief that empathy is an important factor in the treatment process, and my dislike of reading literature or art books unrelated to medical treatment, etc.) categorized into three dimensions: perspective taking, emotional understanding, and empathetic thinking. Each item was evaluated on a Likert scale ranging from 1 to 7, indicating levels of agreement from “completely disagree” to “completely agree.” The cumulative score varied from 20 to 140, with higher scores illustrating enhanced empathy skills. The internal consistency of the scale by *Cronbach’s α* coefficient was 0.750, while the split-half reliability stood at 0.779.

#### 2.4.4 Professional value scores

The professional value scores of participants were collected. The Nurse Professional Values Scale (NPVS) comprises 4 dimensions and 26 items (Frequent self-criticism, protection of patient privacy, etc.), which are categorized into care provision (10 items), activism (8 items), responsibility / freedom / security (5 items), and trust (3 items) (24). Each item was rated on a Likert5 scale, with a total scale score of 260.130 reflecting a higher level of alignment with professional values. The scale demonstrated good internal consistency with a Cronbach’s *α* coefficient of 0.842 and a half-reliability of 0.798.

#### 2.4.5 Professional identity scores

The professional identity scores of participants were collected. The Professional Identity Scale for Nursing Students (PISNS) with 17 items (I have no plans to change my career direction, being a nurse makes me happy, etc.) was used, the questionnaire on nurse professional identity is composed of five aspects: professional self-concept (6 items), retention benefit and turnover risk (4 items), social comparison and self-reflection (3 items), autonomy of career choice (2 items), and social persuasion (2 items) (25). Each question follows a Likert 5-point rating scale, ranging from 1 for “completely inconsistent” to 5 for “completely consistent.” Notably, question 12 uses reverse scoring, while the rest utilize positive scoring. The total score on the questionnaire was 170.85, showing a positive correlation with nurses’ professional identity. The scale demonstrated a Cronbach’s *α* coefficient of 0.827 and a split-half reliability of 0.842.

#### 2.4.6 Training program satisfaction scores

The opinions of intern nurses on various training programs were collected. Satisfaction is determined by a satisfaction survey questionnaire. This survey comprises five key dimensions: content covered in training, instructional techniques utilized, duration of teaching sessions, outcomes of the training, and overall quality of the training program. Participants rated each dimension on a three-point scale ranging from “dissatisfaction” to “satisfied” to “very satisfied.”

### 2.5 Statistical analysis

All data in this study were statistically analyzed using SPSS 21.0 (IBM Corp., Armonk, NY, USA). The Kolmogorov–Smirnov test was performed to assess the normality of the data. Descriptive statistics (*x̅±s*) were used for normally distributed variables. Independent sample *t*-tests were conducted to compare the data before training. Within-group differences were determined by conducting paired sample *t*-tests. Non-normally distributed data were described as *M (P_25_, P_75_)*, with comparisons performed using the Mann–Whitney U test or the Wilcoxon test. Categorical data were presented as *n* (%) and were examined by conducting the *χ^2^* test. Covariance analysis was conducted to compare the parameters after training. Using R-4.4.1 software, univariate logistic regression analysis was conducted to identify factors closely related to the humanistic care ability of intern nurses. All results were considered to be statistically significant at *p* < 0.05.

## 3 Results

### 3.1 Baseline characteristics of intern nurses in the two groups

In this study, 140 intern nurses were included, with 70 participants in each group. The demographic and sociological characteristics of the two groups were similar (*p* > 0.05; Table 2). The average age of the intern nurses in the control group was 24 years (range 23–25 years), whereas in the observation group, it was 25 years (range 24–25 years). The percentage of intern nurses with family members working in healthcare was 44.29% (31/70) in the control group and 40.00% (28/70) in the observation group. Similarly, the percentage of intern nurses who held leadership positions during their university years was 57.14% (40/70) in the control group and 60.00% (42/70) in the observation group (*p* > 0.05, Table 2).

**Table 2:** Comparison of basic information between two groups [M (P_25_, P_75_), *n* (%)].

Index	Control group (*n* = 70)	Observation group (*n* = 70)	Z/χ^2^ value	*p* value
Age (years)	24 (23, 25)	25 (24, 25)	1.910	0.056
Gender			1.527	0.217
Male	18 (25.71)	12 (17.14)		
Female	52 (74.29)	58 (82.86)		
Education level			0.085	0.771
University	63 (90.00)	64 (91.43)		
Graduate student or above	7 (10.00)	6 (8.57)		
Family residence			0.032	0.858
Local	23 (32.86)	24 (34.29)		
Different places	47 (67.14)	46 (65.71)		
Only child			0.310	0.577
Yes	51 (72.86)	48 (68.57)		
No	19 (27.14)	22 (31.43)		
Relatives are medical staff			0.504	0.478
Yes	9 (12.86)	12 (17.14)		
No	61 (87.14)	58 (82.86)		
Student cadre during the university			0.118	0.731
Yes	40 (57.14)	42 (60.00)		
No	30 (42.86)	28 (40.00)		
Reasons for choosing nursing major			0.654	0.721
Independent choice	38 (54.29)	42 (60.00)		
Family requirements	20 (28.57)	19 (27.14)		
Other	12 (17.14)	9 (12.86)		

### 3.2 Changes in the humanistic care ability of intern nurses before and after training

**Figure 2:**
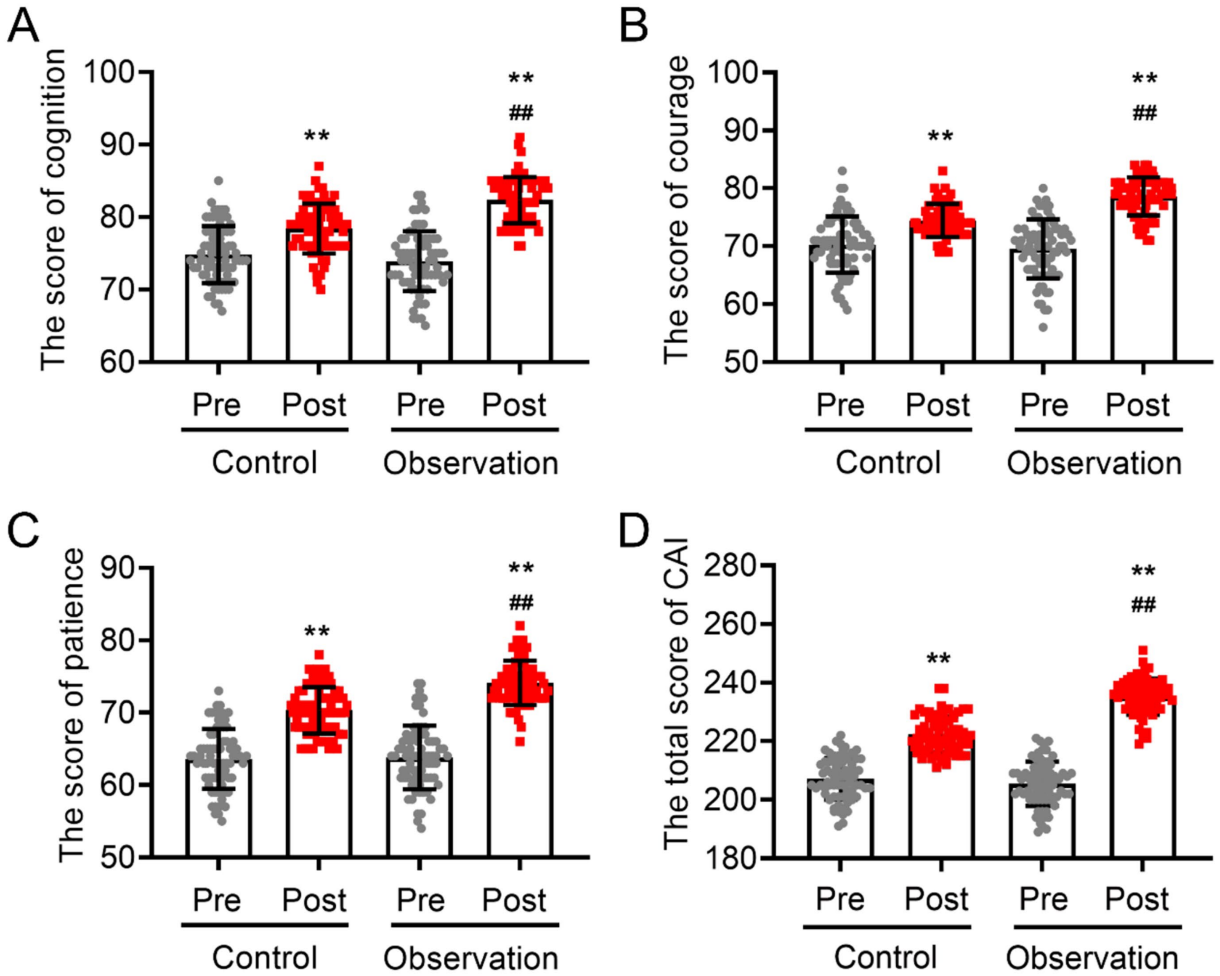
Changes in humanistic care ability of two groups of intern nurses before and after training. **(A)** Cognitive scores, **(B)** courage scores, **(C)** patience scores, and **(D)** total CAI scores. VS pre-training ^**^p < 0.01, VS post-training of the control group ^##^*p* < 0.01.

Before training, no significant differences were found in the scores for cognitive ability, courage, patience, or the total scores of the Care Ability Inventory (CAI) between the two groups of intern nurses (*p* > 0.05; Figure 2). An analysis of each group revealed that after training, there was a notable increase in both the overall scores of humanistic care ability (in the control group: 222.34 ± 6.13 after training vs. 207.14 ± 7.10 before training; in the observation group: 235.21 ± 6.18 after training vs. 205.47 ± 7.54 before training) and the scores of individual subscales across all groups (*p* < 0.05). The results of covariance analysis post-training revealed a significant difference between the observation group and the control group in subscale of their total CAI score (235.21 ± 6.18 in the observation group vs. 222.34 ± 6.13 in the control group), cognitive score (82.34 ± 3.18 in the observation group vs. 78.43 ± 3.44 in the control group), courage score (78.60 ± 3.29 in the observation group vs. 74.46 ± 2.85 in the control group), and patience subscale score (74.11 ± 3.06 in the observation group vs. 70.33 ± 3.20 in the control group) (*p* < 0.05; Figure 2). These findings suggest that practical training based on narrative nursing can more effectively and comprehensively improve the humanistic care ability of intern nurses.

### 3.3 Differences in empathy ability between the two groups of intern nurses after training

**Figure 3:**
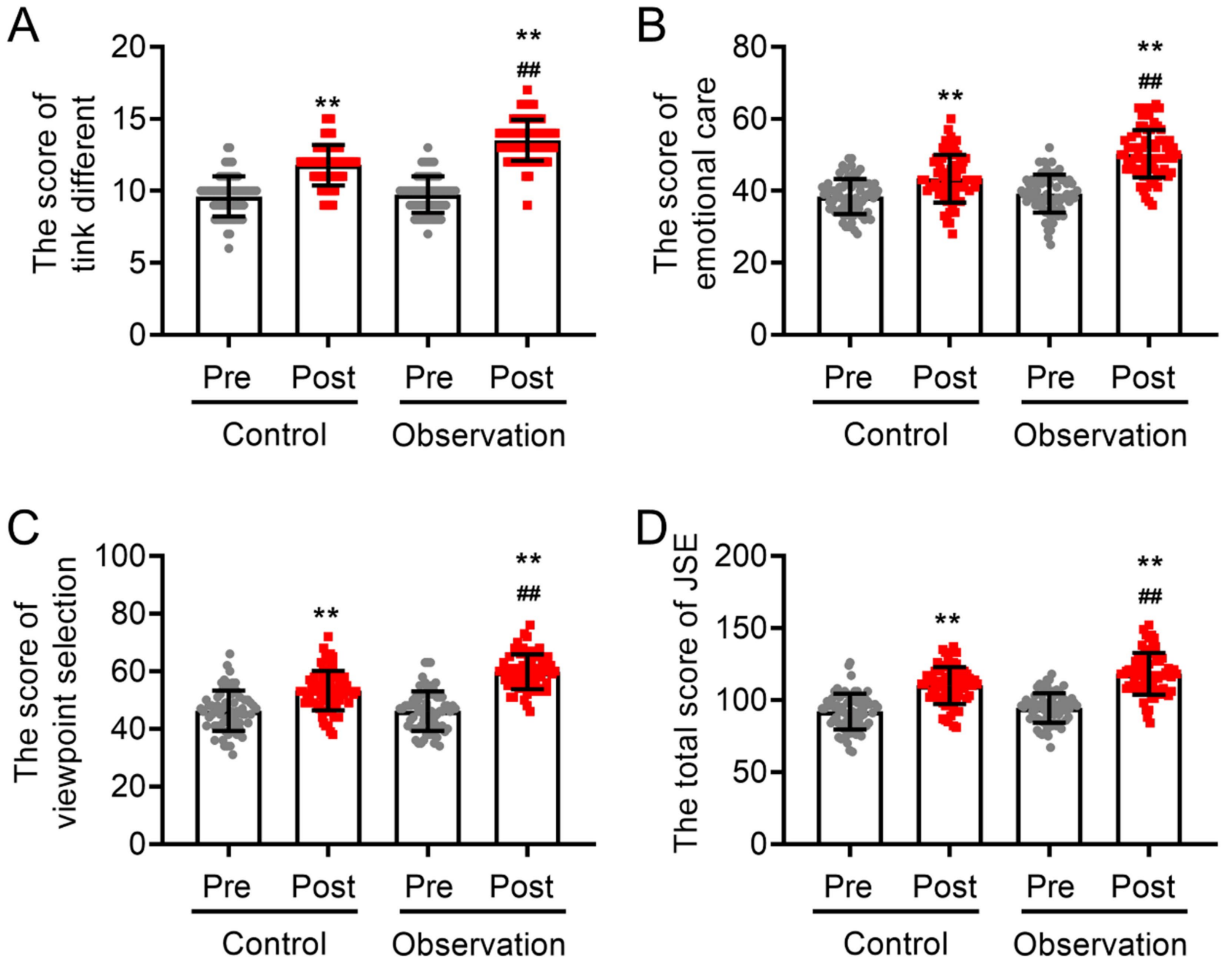
Scores of empathy ability and related items among intern nurses with and without practical training based on narrative nursing. **(A)** Perspective-taking scores, **(B)** emotional care scores, **(C)** viewpoint selection scores, and **(D)** total scores of JSE. VS pre-training ^**^p < 0.01, VS post-training of the control group ^##^*p* < 0.01.

At baseline, no statistically significant differences were found in the initial total JSE score between the two sets of intern nurses (*p* > 0.05; Figure 3). The three items of the JSE (thinking differently, emotional care, and viewpoint selection) yielded similar results to the total score of the JSE (p > 0.05; Figure 3). However, after 6 months, the total score (in the control group: 110.10 ± 12.86 after training vs. 92.04 ± 12.38 before training; in the observation group: 118.29 ± 14.53 after training vs. 94.54 ± 10.28 before training) and the scores of the three items for both groups significantly improved after training (*p* < 0.05). The analysis of covariance revealed that the observation group presented a noticeably greater increase in empathy-related domain scores compared to the control group post-training (118.29 ± 14.53 in the observation group vs. 110.10 ± 12.86 in the control group). Specifically, the scores for think different (13.51 ± 1.42 in the observation group vs. 111.79 ± 1.41 in the control group), emotional care (50.33 ± 6.60 in the observation group vs. 43.40 ± 6.62 in the control group), and viewpoint selection (59.83 ± 6.07 in the observation group vs. 53.26 ± 6.86 in the control group) were significantly greater than those of the control group (all *p* < 0.05; Figure 3). These findings suggest that the instruction provided to the observation group may have had a stronger effect on their professional development and caregiving abilities.

### 3.4 Differences in the professional values of intern nurses with different training methods

**Figure 4:**
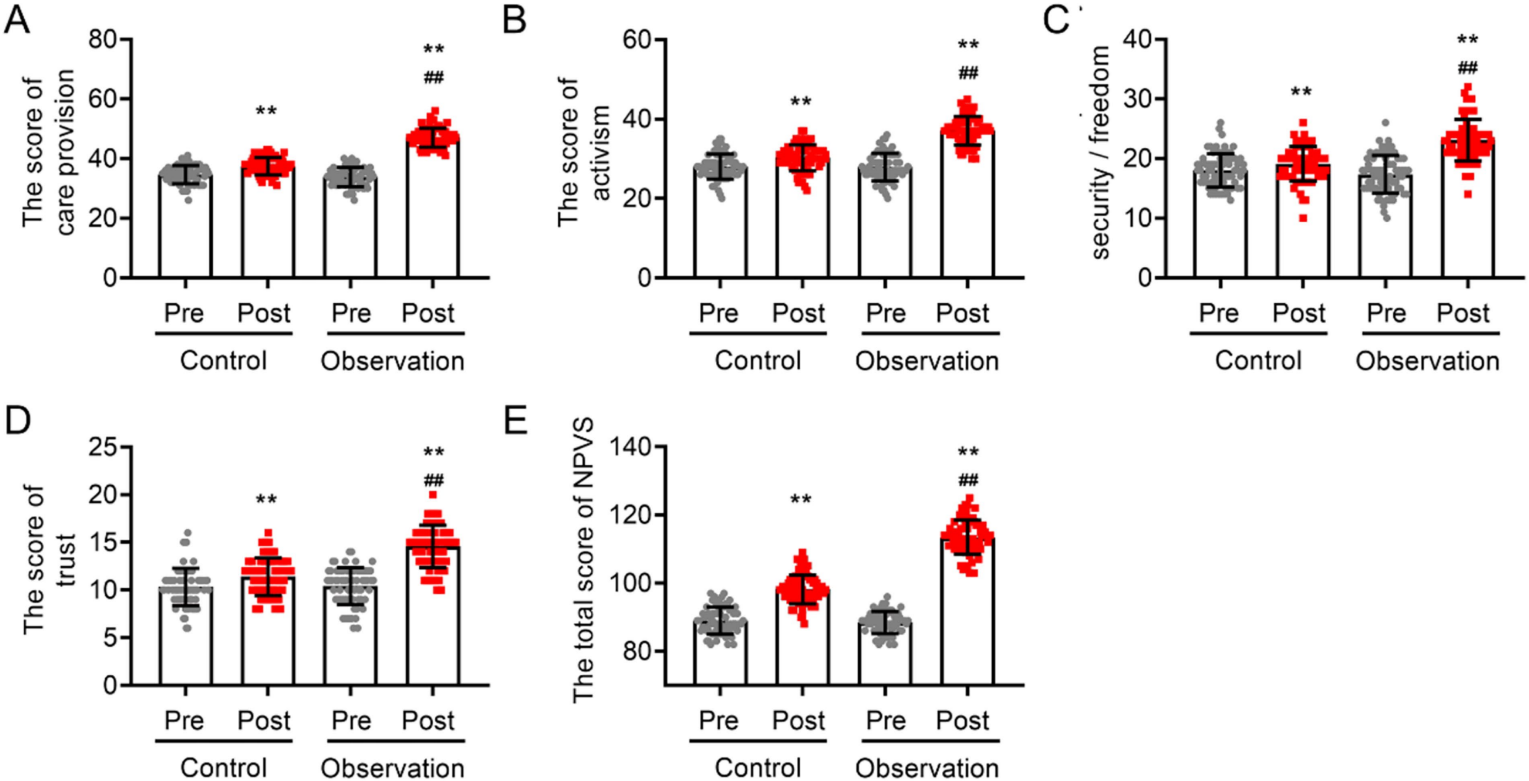
The influence of narrative nursing-based practical training on professional values of intern nurses. **(A)** Care provision scores, **(B)** activism scores, **(C)** responsibility/security/freedom scores, **(D)** trust scores, and **(E)** total NPVS scores. VS pre-training ^**^p < 0.01, VS post-training of the control group ^##^*p* < 0.01.

Before the training, no statistically significant difference was observed in the total scores of the NPVS or the four items (care provision, activism, responsibility/security/freedom, and trust) between the two groups (*p* > 0.05; Figure 4). The results of the within-group analysis revealed a significant improvement in the professional value scores of patients in both groups after 6 months of training (*p* < 0.05). After 6 months of training, the analysis of covariance indicated that the scores for the observation group were significantly higher than those for the control group in the areas of care provision (47.00 ± 3.19 in the observation group vs. 37.44 ± 2.91 in the control group), activism (37.06 ± 3.60 in the observation group vs. 30.27 ± 3.23 in the control group), responsibility/safety/freedom (23.07 ± 3.47 in the observation group vs. 19.11 ± 2.90 in the control group), trust (14.57 ± 2.22 in the observation group vs. 11.40 ± 1.97 in the control group), and the overall professional values score (113.46 ± 5.01 in the observation group vs. 98.14 ± 4.28 in the control group) (*p* < 0.05; Figure 4). These findings suggest that the use of practical training rooted in narrative nursing can more effectively foster the professional values of intern nurses.

### 3.5 Comparison of professional identity between the control group and observation group

**Figure 5:**
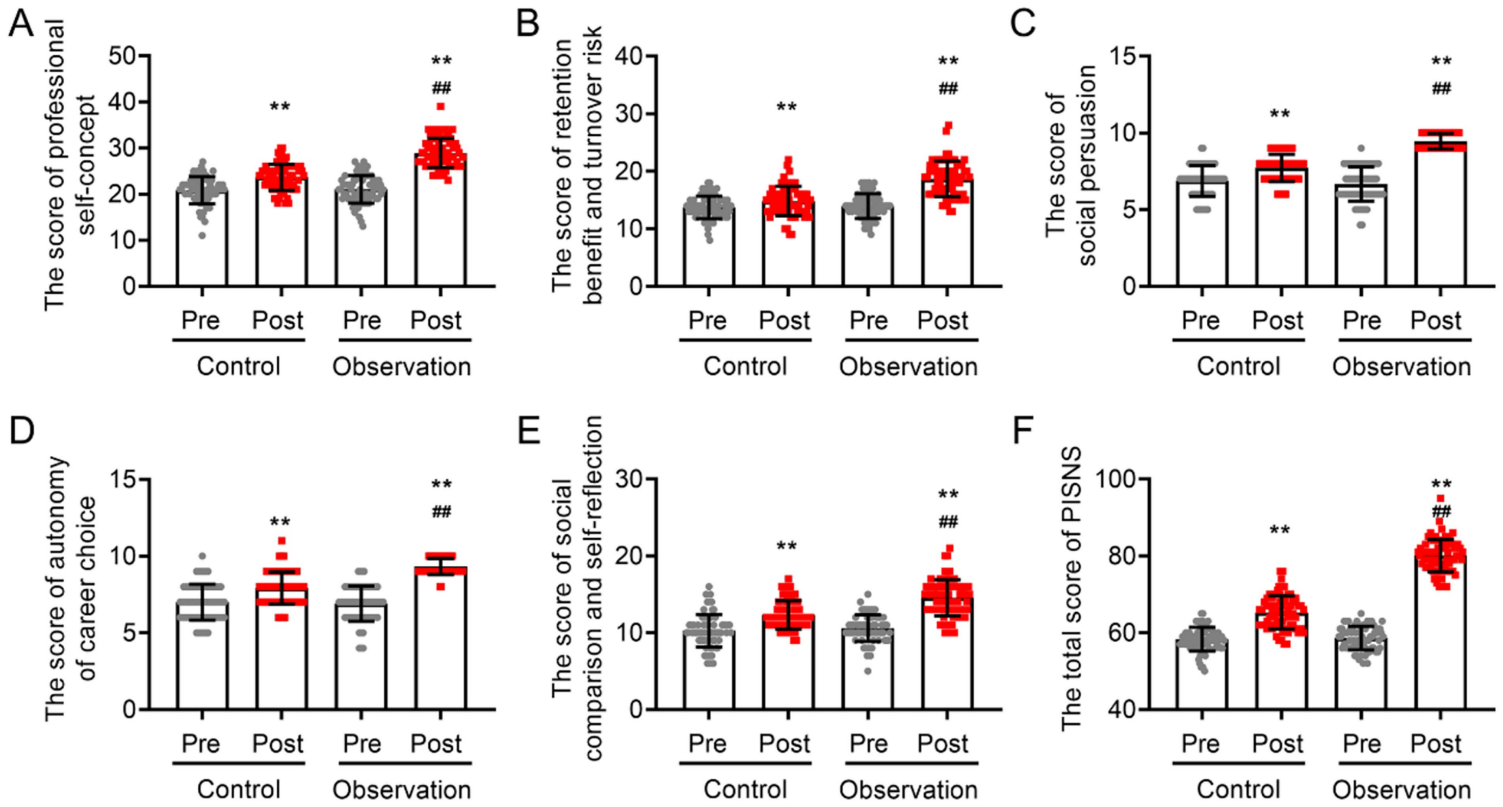
Comparison of professional identity between observation group and control group before and after training. **(A)** Care professional self-concept scores, **(B)** retention benefit and turnover risk scores, **(C)** social comparison scores, **(D)** autonomy of career choice scores, **(E)** social persuasion and self-reflection scores, and **(F)** total PISNS scores. VS pre-training ^**^p < 0.01, VS post-training of the control group ^##^*p* < 0.01.

Before training began, the two groups had similar PISNS scores and five items (professional self-concept, retention benefit and turnover risk, social comparison and self-reflection, autonomy of career choice, and social persuasion) (*p* > 0.05; Figure 5). However, both training regimens significantly increased the PISNS score and five-item score (*p* > 0.05, Figure 5). An analysis of covariance was performed, and the results indicated that following the adjustment for influencing factors, the intern nurses in the observation group presented significantly higher scores across all dimensions of professional identity, including “professional self-concept (28.91 ± 3.15 in the observation group vs. 23.67 ± 2.87 in the control group),” “advantages and disadvantages of remaining vs. departing (18.67 ± 3.06 in the observation group vs. 14.83 ± 2.55 in the control group),” “social comparison and self-reflection (14.57 ± 2.35 in the observation group vs. 12.31 ± 1.85 in the control group),” “independence in career selection (9.31 ± 0.53 in the observation group vs. 7.91 ± 1.05 in the control group),” and “social persuasion (9.46 ± 0.50 in the observation group vs. 7.71 ± 0.89 in the control group),” as well as the overall score (80.07 ± 4.20 in the observation group vs. 65.30 ± 4.33 in the control group), compared to those in the control group (*p* < 0.05; Figure 5).

### 3.6 Satisfaction of intern nurses with different training methods

After 6 months of training, a satisfaction survey was conducted with all intern nurses. The results revealed that intern nurses who received narrative-based practical training had a satisfaction rate of 91.42% (64/70), which was significantly higher than the satisfaction rate of 78.57% (55/70) reported by the control group (*p* < 0.05; Table 3). Moreover, intern nurses in the observation group rated various aspects of the training program, such as content, methods, duration, outcomes, and overall effectiveness, as “very satisfied” at significantly higher levels compared to the control group (all p < 0.05; Table 3). These findings suggest that the narrative-based practical training method may be more effective in engaging and motivating intern nurses to learn.

**Table 3:** Satisfaction of intern nurses for practical training based on narrative nursing [*n* (%)].

Index	Control group (*n* = 70)	Observation group (*n* = 70)	χ^2^ value	*p* value
Training content			2.032	0.042
Dissatisfaction	11(15.71)	4(5.71)		
Satisfied	6(8.57)	4(5.71)		
Very satisfied	53(75.72)	62(88.58)		
Training methods			1.985	0.047
Dissatisfaction	7(10.00)	3(4.29)		
Satisfied	10(14.28)	5(7.13)		
Very satisfied	53(75.72)	62(88.58)		
Teaching schedule			2.048	0.041
Dissatisfaction	5(7.13)	2(2.87)		
Satisfied	11(15.71)	5(7.13)		
Very satisfied	54(77.16)	63(90.00)		
Training results			2.145	0.032
Dissatisfaction	4(5.71)	5(7.13)		
Satisfied	15(21.43)	3(4.29)		
Very satisfied	51(72.86)	62(88.58)		
Overall training program			2.007	0.038
Dissatisfaction	3(4.29)	2(2.87)		
Satisfied	12(17.14)	4(5.71)		
Very satisfied	55(78.57)	64(91.42)		

### 3.7 Identifying factors affecting humanistic care ability

**Figure 6:**
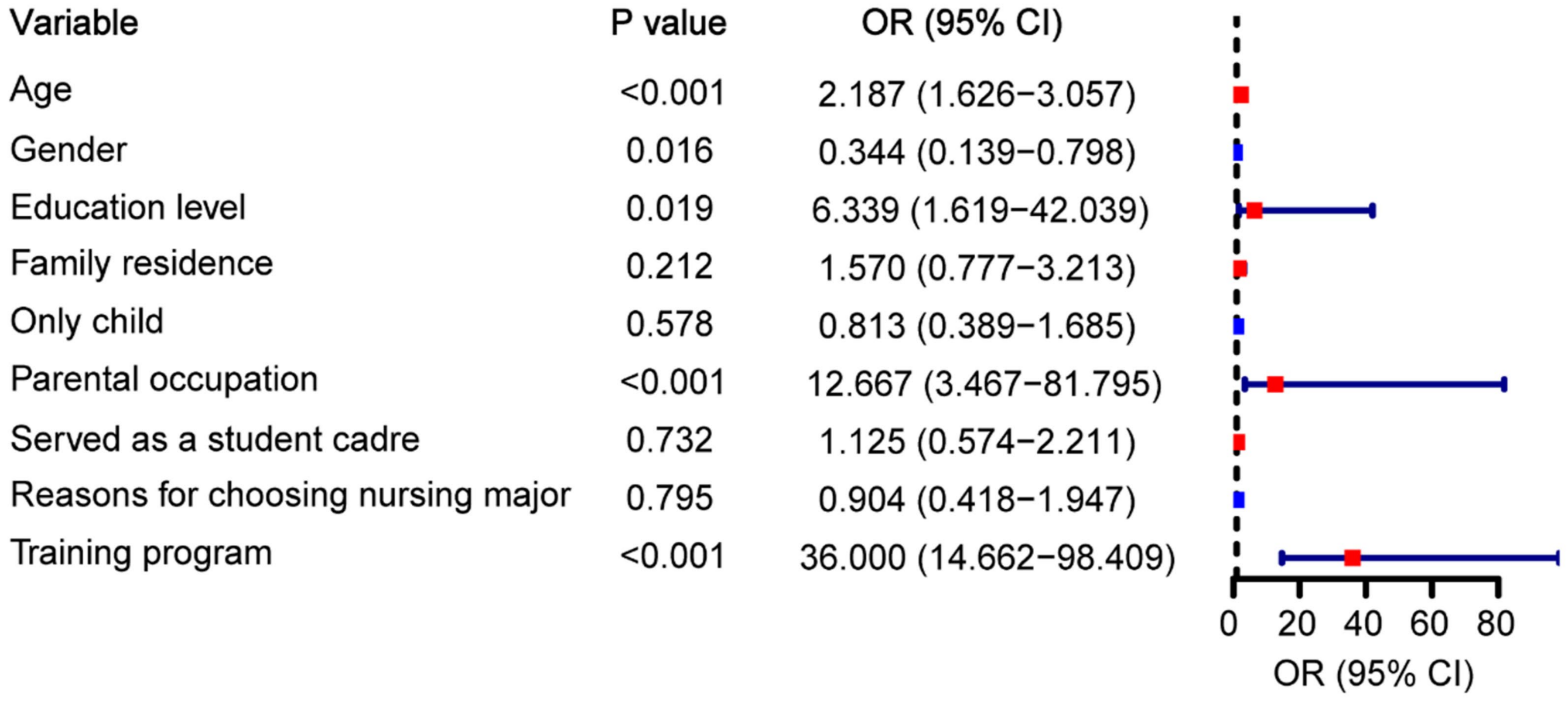
Univariate logistic regression analysis to identify the influencing factors of humanistic care ability.

After training, the participants were categorized into two groups: a high-ability group (CAI score > 230; *n* = 70) and a low-ability group (CAI score ≤ 230; *n* = 70) based on the median CAI score. The humanistic care ability and associated characteristics of intern nurses were then evaluated by assigning them values. Univariate logistic regression analysis was conducted by training methods and clinicopathological factors such as age, gender, education level, family residence, only child status, parental occupation, serving as a student cadre, and reasons for choosing a nursing major. The variable assignment of the regression analysis is shown in Table 4. The results of univariate logistic regression analysis revealed that age, gender, education level, parental occupation, and training program were significantly related to the humanistic care ability of the intern nurses (*p* < 0.05; Figure 6).

**Table 4:** Variable assignment of regression analysis.

Index		Variable assignment
Gender	Male	1
	Female	0
Education level	University	0
	Graduate student or above	1
Family residence	Local	1
	Different places	0
Only child	Yes	1
	No	0
Parental occupation	Relatives are medical staff	1
	Others	0
Served as a student cadre	Yes	1
	No	0
Reasons for choosing nursing major	Family requirements	1
	Independent choice	2
	Others	3
Training program	Regular training	0
	Practical training based on narrative nursing	1
Outcome	High-ability group	1
	Low-ability group	0

## 4 Discussion

The concept of narrative education has received considerable attention from researchers in recent years and has emerged as a focal point in the realm of humanistic care (21, 22). The humanistic care education in China has started recently; therefore, the humanistic care capabilities of nurses in China need to be further improved to adapt to the highly stringent professional requirements and the clinical needs of patients. This deficiency is attributed to the insufficient emphasis on nurturing humanistic care abilities in nursing interns during their clinical training and education (23–25). In this study, we retrospectively analyzed the significance of using narrative nursing-based practical training to improve the humanistic care ability of intern nurses. The findings indicated a notable improvement in the humanistic care scores of intern nurses who underwent narrative nursing-based practical training. The intern nurses with narrative nursing-based practical training presented considerably higher scores for various professional values and professional identities. An evaluation of the changes in empathy and narrative abilities among both groups of intern nurses before and after training revealed that the intern nurses in the observation group had significantly greater empathy and narrative abilities than the pretraining and control groups. Additionally, satisfaction surveys revealed that intern nurses were more satisfied with narrative nursing-based practical training. To further assess the relationship between this practical training and the humanistic care competencies of intern nurses, logistic regression analysis was conducted, and the results supported the notion that implementing narrative nursing-based practical training significantly improves humanistic care competencies. This study provides evidence support for nursing humanities education, confirming that narrative nursing practice training is an effective means to enhance interns’ core competencies, and helps guide medical institutions to optimize training strategies, ultimately benefiting patients through improving nursing quality.

Nursing interns serve as the backup workforce for nursing tasks, and their ability to provide humanistic care is directly linked to the quality of nursing work (26). The medical college emphasizes teaching related knowledge in humanistic care theory, but lacks the cultivation of practical caring skills. Additionally, during clinical practice, teachers prioritize guiding clinical nursing operations rather than instructing humanistic care. Providing training in humanistic care during clinical practice can help nursing interns apply these skills in their work and develop good habits. Previous studies have suggested narrative nursing, which has been successfully applied in geriatric and community nursing, as well as in the development of the humanistic qualities of nurses. This approach not only forms a bridge between theory and practice but also helps enhance and nurture the caring abilities of nurses (27–29). Xue et al. investigated the effect of narrative medicine theory education and narrative writing on humanistic care ability based on a network platform. The results showed that it can promote the development of the professional quality, empathy, and humanistic care ability of nursing undergraduates (22). Moreover, Guo et al. reported that narrative nursing training using digital storytelling effectively enhances the humanistic qualities of intensive care unit nursing students (30). Although the breadth and depth of the application of narrative education research in nursing teaching in China are continuously increasing, there are certain limitations, including the lack of diversity in application forms and implementation methods, which cannot fully ensure the accuracy and usability of materials. In our study, the narrative nursing-based practical training implemented is characterized by various forms. This approach helps merge theoretical training with practical experience, ultimately improving the humanistic care proficiency of nursing interns, including cognitive scores, courage scores, patience scores, and total CAI scores. Although routine training can improve the level of humanistic care provided by nursing interns, including cognitive scores, courage scores, patience scores, and CAI total scores, practical training based on narrative nursing can more effectively improve the above indicators. These findings indicate that narrative-based nursing is better than conventional training. In regular training, the organization and management, environment and facilities of hospital nursing humanistic care provide interns with a comprehensive knowledge framework. This systematic learning helps them understand the multi-dimensional connotation of humanistic care. Importantly, the implementation method of practical training based on narrative nursing in our study was more diverse, using a combination of online and offline learning to conduct training. It not only included teaching based on videos but also situational rehearsals, book salons, and reflective diaries. Improving humanistic care ability enables nursing interns to listen to the narratives of patients, understand their situations, and empathize with them. While previous studies focused on how training in narrative nursing or humanistic care can improve the skills of clinical nurses and patient care outcomes, this study focused on the training process of nursing interns. Reflective diaries in narrative nursing practice training can encourage nursing interns to record their stories with patients, which deepens the internal connection between nursing interns and patients, enhances their self-reflection, clarifies their problems, and helps them solve them, thereby improving their ability to provide humanistic care. In subscale of scenario exercises, the nursing interns were allowed to further hone their nursing and communication skills. By perceiving the caring atmosphere, nursing interns can further understand the feelings of patients and enhance patient-centered concepts, thereby improving the humanistic care abilities of students. Through scenario exercises, reading salons, and reflecting on the form of diaries, this study fills the research gap in narrative medicine in the “online and offline integration mode” of nursing education, provides a theoretical basis for the application of narrative medicine in improving the humanistic care ability of nursing interns, and helps reduce the occurrence of conflicts between doctors and patients.

Modern medicine is advancing rapidly, with many healthcare workers focusing on treating patients using advanced technology. This means that they may be neglecting the demands of patients and their emotions. According to Galuska et al., narrative nursing training, when implemented as a standalone approach, provides a more efficient way for intern nurses to realistically reflect on their work and personal experiences (31). Maringgele et al. highlighted the importance of altruistic behavior in enhancing the empathy skills of intern nurses (32). Based on the results of the studies mentioned above, we discovered that practical training using narrative nursing significantly improved the overall empathy ability scores and scores for each item among nursing interns, as determined by the JSE score. We also found that practical training involving narrative nursing improved JSE scores more significantly than conventional training. This finding is similar to the findings of Chen et al. (33) and indicates that practical training based on narrative nursing is more effective than regular training in improving empathy skills among nursing interns and is worth promoting among healthcare professionals. In regular training, clinical instructors can use role-playing or case analysis to have interns consider issues from the perspective of patients, thereby enhancing their understanding of patients’ emotions. Through repeated practice and guidance, interns can more sensitively perceive patients’ needs and respond in a more considerate manner, thereby improving their empathy skills. Notably, the narrative nursing-based practical training is even more diverse. For the narrative nursing-based practical training, watching videos and reading salons, nursing interns actively observe the humanistic behaviors of clinical medical staff and, through description and reflection, actively assess the effect and value of these behaviors, ultimately becoming new behaviors. Watching videos and reading salons can trigger deep empathy among nurses toward the situation of patients, guide them to think from their perspective, increase their emotional experiences through structured cognition, and ultimately improve the empathy ability of nursing interns. The improvement in the empathy ability score highlighted the importance of narrative nursing in improving humanistic care ability. It can enable nursing interns to communicate better with patients and improve patient compliance. Moreover, identifying ways to maintain the steady growth of empathy through continuous training in the future is crucial, and it is also the focus of follow-up research.

As the driving force of the medical industry, the professional identity of nursing interns not only affects their current acquisition of medical knowledge and skills but also plays a vital role in their future careers as they strive to become doctors. Compared to those before the training, the professional identities and professional values of the two groups of nursing interns were significantly enhanced after the training, and the nursing interns who received practical training based on narrative nursing presented a more significant enhancement in professional identity and professional values. This finding indicates that while regular training can improve professional identity and professional values, the advantages of practical training based on narrative nursing are more apparent and may help replace regular training. During the regular training, the clinical instructors, as practitioners of humanistic care, demonstrated the value of the nursing profession through their words and actions, enhancing the interns’ sense of identification with nursing work. During the training, the interns experienced the humanistic nature of the nursing industry and understood the core value of nursing work, thereby enhancing their professional values. Moreover, the practical training based on narrative nursing encompasses various forms. Watching the video “Is there temperature in nursing?” This allows nursing interns to understand and feel the importance and honor of nurses in the medical system and save lives, and also enables them to admire and feel a sense of honor in the nursing profession. The professional stories of excellent nurses in videos can inspire the exemplary spirit of nursing interns, which can further improve their sense of professional identity. When watching videos, being more proactive in paying attention to “value details” forms a continuous growth cycle of “input-reflection-output,” ultimately shifting from “passively accepting career positioning” to “actively defining career meaning.” This further defines the professional identity and improves the values of nursing interns. A strong sense of professional identity and values not only assists nursing interns in maintaining a positive psychological state while practicing nursing but also increases their enthusiasm for work and learning, helping them develop correct professional values and reducing staff turnover. Practical training that is based on narrative nursing allows for reflection and adjustment of methods and techniques based on feedback from practical results. This training not only generates positive experiences and effects but also encourages nursing interns to actively reflect on themselves and consider their relationships with patients in a busy clinical setting. This approach not only focuses on diagnosis and treatment but also emphasizes internal cognitive recognition. We also found that intern nurses who underwent practical training rooted in narrative nursing reported significantly higher levels of satisfaction. This highlights the positive effect of narrative nursing-based practical training on enhancing the professional values and sense of identity of nursing interns.

To further investigate the effect of receiving narrative nursing-based practical training on the performance of intern nurses, we conducted correlation analysis by conducting univariate logistic regression analysis. Our results confirmed that practice training based on narrative nursing was the key factor in improving the humanistic care ability of practice nurses. This provides strong evidence for the role of practical training based on narrative nursing in enhancing the humanistic care ability of intern nurses. Furthermore, intern nurses from May 2022 to May 2023 received regular training, whereas intern nurses from May 2023 to May 2024 received practical training in narrative nursing. The evaluation of outcomes by intern nurses, who know what kind of training they have received, should be avoided. Therefore, self-reinforcing awareness (such as the Hawthorne effect) does not affect the improvement of indicators, and the experiment is objective. These findings promote education reform in the nursing discipline, bridge the gap in the humanistic education of intern nurses in clinical practice, and provide a reference for promoting the development of medical humanistic quality.

This study had certain limitations. First, the use of only intern nurses from a single center may have introduced bias into the results. Therefore, a multicenter study is needed to confirm these findings. Second, although the narrative nursing practical training in this study was conducted for 6 months, most intern nurses typically participated in clinical practice for more than a year. It is uncertain whether the ability to provide humanistic care decreases after the training period, which necessitates further investigation. Third, assessing the impact of narrative nursing-based practical training on intern nurses of different genders or educational backgrounds requires additional research based on hierarchical analysis to determine whether the effects are consistent across various groups.

## 5 Conclusion

To summarize, this study showed that practical training based on narrative nursing, which combines online and offline learning methods, including situational drills, reading salons, and reflective diaries, can effectively enhance the humanistic care ability, empathy ability, professional values, and professional identity of nursing interns. Compared to those who received regular training, nursing interns who received practical training based on narrative nursing showed more significant improvements in the above indicators. By enhancing the humanistic care and empathy capabilities of nursing interns, narrative nursing-based practical training not only advances the theoretical framework of nursing humanistic education but also lays a foundation for developing a localized nursing humanistic education system. Moreover, it enriches the medical experience of patients, thereby increasing their overall satisfaction with healthcare services. Fostering professional values and identity through narrative practice allows nursing interns to perceive the significance of their roles, strengthens their professional convictions, and helps mitigate talent attrition in the nursing field.

## Data Availability

The original contributions presented in the study are included in the article/supplementary material, further inquiries can be directed to the corresponding author.
